# Identification of a Seven-lncRNA-mRNA Signature for Recurrence and Prognostic Prediction in Relapsed Acute Lymphoblastic Leukemia Based on WGCNA and LASSO Analyses

**DOI:** 10.1155/2021/6692022

**Published:** 2021-06-09

**Authors:** Haiyan Qi, Long Chi, Xiaogang Wang, Xing Jin, Wensong Wang, Jianping Lan

**Affiliations:** ^1^Department of Hematology, Zhejiang Provincial People's Hospital, People's Hospital of Hangzhou Medical College, Hangzhou 310014, China; ^2^Department of ICU, Zhejiang Greentown Cardiovascular Hospital, Hangzhou 310012, China

## Abstract

Abnormal expressions of long noncoding RNAs (lncRNAs) and protein-encoding messenger RNAs (mRNAs) are important for the development of childhood acute lymphoblastic leukemia (ALL). This study developed an lncRNA-mRNA integrated classifier for the prediction of recurrence and prognosis in relapsed childhood ALL by using several transcriptome data. Weighted gene coexpression network analysis revealed that green, turquoise, yellow, and brown modules were preserved across the TARGET, GSE60926, GSE28460, and GSE17703 datasets, and they were associated with clinical relapse and death status. A total of 184 genes in these four modules were differentially expressed between recurrence and nonrecurrence samples. Least absolute shrinkage and selection operator analysis showed that seven genes constructed a prognostic signature (including one lncRNA: LINC00652 and six mRNAs: INSL3, NIPAL2, REN, RIMS2, RPRM, and SNAP91). Kaplan-Meier curve analysis observed that patients in the high-risk group had a significantly shorter overall survival than those of the low-risk group. Receiver operating characteristic curve analysis demonstrated that this signature had high accuracy in predicting the 5-year overall survival and recurrence outcomes, respectively. LINC00652 may function by coexpressing with the above prognostic genes (INSL3, SNAP91, and REN) and lipid metabolism-related genes (MIA2, APOA1). Accordingly, this lncRNA-mRNA-based classifier may be clinically useful to predict the recurrence and prognosis for childhood ALL. These genes represent new targets to explain the mechanisms for ALL.

## 1. Introduction

Acute lymphoblastic leukemia (ALL) which results from the clonal proliferation of immature T- or B-lymphoid cells in the bone marrow is the most common malignant hematologic disorder in childhood, accounting for approximately 35% of all childhood malignancies [1, 2]. Chemotherapy is the main therapeutic approach for childhood ALL, with the rate of complete remission (CR) being achieved higher than 90% [3]. However, 10% of patients still will experience relapse, leading to their eventual death [3, 4]. Thus, it is considerably essential to early identify cases at a high risk of relapse and predict their overall survival (OS) to schedule more individualized treatments.

Recently, the rapidly developed molecular technique has led to an expansion of knowledge regarding the pathogenesis of diseases, and several molecular biomarkers have been suggested to predict the relapse and the prognostic outcomes for cancers [5–8], including ALL. For example, Jia et al. [9] identified that the midkine gene (MK) may be a possible prognostic marker. The expression of MK was significantly higher in patients with relapsed ALL than those with CR or at diagnosis. Patients with highly expressed MK harbored poor OS (*p* = 0.022) and relapse-free survival (RFS, *p* = 0.047) compared with patients with low expression of MK. Weng et al. [10] found that the mRNA level of the TP53 gene was significantly higher in patients with ALL than controls. Multivariate analysis revealed that highly expressed TP53 was an independent factor for the prediction of poor OS and RFS (*p* < 0.001). Sharaf-Eldein et al. [11] demonstrated that macrophage migration inhibitory factor (MIF) expressers had a significantly lower incidence of CR, a higher incidence of relapse, and shorter OS and disease-free survival (DFS) than low MIF expressers. By analysis of high-throughput microarray data in the Therapeutically Applicable Research to Generate Effective Treatments (TARGET) database and European Bioinformatics Institute (EMBL-EBI, accession number E-MTAB-1216), Jing and Li [12] screened 59 genes to be significantly associated with RFS in pediatric B-ALL. Using self-collected bone marrow specimens and microarray data, Cleaver et al. [13] modeled a five-gene classifier that could accurately predict the relapse risk (82% for the training dataset and 79% for the validation dataset) and worse RFS (*p* < 0.05) for pediatric T-ALL patients. However, recurrence and prognostic biomarkers for relapsed childhood ALL remain rarely reported.

In addition to protein-encoding messenger RNAs (mRNAs), abnormal expressions of long noncoding RNAs (lncRNAs) have also been proved as important mechanisms for the development of childhood ALL [14, 15]. For example, Ouimet et al. [16] identified that lncRNA RP11-137H2.4 was overexpressed in childhood B-ALL samples, as compared to B-cells isolated from human cord blood. Silencing of RP11-137H2.4 significantly reduced the proliferation and migration but increased the apoptosis and glucocorticoid sensitivity of childhood ALL cell lines (Reh and NALM-6), which may be related to its regulation effects on the downstream MAPK and cell cycle pathways [16]. Wang et al. [17] found that lncRNA NALT was upregulated in the bone marrow of childhood T-ALL compared with age-matched volunteers. NALT promoted T-cell proliferation and stimulated a tumor formation in a murine xenograft model by interacting with NOTCH1 to increase its transcription [17]. Transcriptome analysis of bone marrow T-cells from childhood T-ALL identified that lnc-INSR was highly expressed. Function assays revealed that lnc-INSR promoted Treg distribution and decreased the percentage of cytotoxic T lymphocytes, consequentially inducing tumor growth [18]. These findings indicated that lncRNAs may also have the prognostic potential for childhood ALL, however, which had not been explored previously.

In this study, we aimed to develop and validate a new signature for the prediction of recurrence and prognosis in relapsed childhood ALL patients with any immunophenotype by integrating the expression profiles of protein-encoding mRNAs and lncRNAs. Furthermore, the regulatory mechanisms between lncRNAs and mRNAs were also predicted to reveal the underlying functions of the identified signature.

## 2. Materials and Methods

### 2.1. Data Resources and Processing

Transcriptome and clinical data of ALL patients were extracted from the TARGET database (https://ocg.cancer.gov/programs/target, updated December 1, 2019). Among them, 105 samples (including 13 recurrence and 92 nonrecurrence; including B-cell and T-cell) were included in this study because the recurrence and survival information were provided in these samples. Furthermore, the EMBL-EBI ArrayExpress database (https://www.ebi.ac.uk/arrayexpress/) was also searched using the key words “acute lymphoblastic leukemia” and “paediatric/child” to identify other transcriptome datasets. The datasets were included if they met the following inclusion criteria: (1) expression profiles were analyzed, (2) the bone marrow samples at relapse or diagnosis were obtained for analysis of ALL, (3) the number of samples (including the recurrence and nonrecurrence comparison) was more than 50, and (4) survival outcomes were provided. As a result, three microarray datasets were enrolled according to the inclusion criteria of (1)-(3), including GSE60926 (*n* = 50, including 28 recurrence and 22 nonrecurrence; all were B-cell precursor ALL), GSE28460 (*n* = 98, including 49 recurrence and 49 nonrecurrence; all were B-cell precursor ALL), and GSE17703 (*n* = 101, including 11 recurrence and 90 nonrecurrence; including B-ALL and T-ALL) which were used for module validation analysis, while two microarray datasets were enrolled because they satisfied the inclusion criteria of (1), (2), and (4), including E-MTAB-1216 (*n* = 80, including 23 recurrence and 57 nonrecurrence; including B-ALL and T-ALL) and E-MTAB-1205 (*n* = 50, including 21 recurrence and 29 nonrecurrence; all were T-ALL) which were used for survival validation analysis. This integrated study was a second analysis of public data; thus, patient consent was not required.

The known mRNAs and lncRNAs in each transcriptome dataset were reannotated by the HUGO Gene Nomenclature Committee (HGNC; http://www.genenames.org/) that includes the standard nomenclature for 4,527 lncRNAs and 19,201 protein-coding genes [19]. Only the shared mRNAs and lncRNAs in all included datasets were used for the following analyses.

### 2.2. Identification of Crucial Module RNAs in ALL

The weighted gene coexpression network (WGCNA) [20] is a network biology method that can partition the genes into different coexpression modules in which the genes were considered to be highly interconnected. Thus, construction of WGCNA may facilitate the identification of hub genes for the development of ALL. In the present study, ALL-related modules were screened via the WGCNA package in R (version 1.61; https://cran.r-project.org/web/packages/WGCNA/index.html) based on the training dataset of TARGET and validation datasets of GSE60926, GSE28460, and GSE17703. First, the correlations in the expression and connectivity of RNAs among the four datasets were analyzed to ensure their comparability. Second, a soft-thresholding power (*β*) was set from 1 to 30 by using the pickSoftThreshold function to calculate *R*^2^ of the logarithm of the node connection log(*k*), and the logarithm of the probability of *k* is log(*p*(*k*)). The corresponding *β* was selected when *R*^2^ reached 0.9 for the first time, which means that the network at this time may follow the scale-free topology criteria. Third, the adjacency matrix was transformed into a topological overlapping matrix to construct the network, and the gene dendrogram was established according to the dissimilarity among different RNAs. Fourth, highly correlated coexpression gene modules were identified using the DynamicTreeCut method, with the threshold set as cutHeight = 0.995 and minSize = 100. Fifth, the stability of modules screened from training datasets was determined by the modulePreservation statistics using the other three validation datasets. A preservation *Z*-score larger than 5 indicated that the module was stable, and the coexpressed relationships identified in the training dataset also can occur in the validation set. Sixth, the potential functions of stable modules were annotated using the userListEnchment function. Seventh, the associations between module eigengenes (ME, representing the expression profiles of module genes) and clinical features were analyzed according to Pearson's correlation test.

### 2.3. Identification of Differential RNAs in Relapsed ALL

The differentially expressed RNAs (DERs) between recurrence and nonrecurrence samples of four datasets (TARGET, GSE60926, GSE28460, and GSE17703) were identified using the MetaDE.ES function in MetaDE package (version 1.0.5, https://cran.r-project.org/web/packages/MetaDE/). First, the expression heterogeneity of each RNA in four datasets was assessed by tau^2^ statistic and chi-square-based *Q*-test. Only the RNAs with no heterogeneity (tau^2^ and *Qp* value > 0.05) were included. Next, the difference of each RNA between recurrence and nonrecurrence was calculated, with the false discovery rate (FDR) < 0.05 set as the cut-off point. Furthermore, the log_2_FC (fold change) of each RNA in each dataset was also computed. Only the RNAs with a consistent differential trend in four datasets were selected.

### 2.4. Identification of a Prognostic Signature

The overlap between stable module RNAs and differentially expressed RNAs was obtained as the seed for screening the prognostic signature. Using the samples in the TARGET dataset (training), univariate Cox regression analysis was first performed to preliminarily screen the RNAs associated with OS using the “survival” package in R (version, 2.41-1; http://bioconductor.org/packages/survivalr/). Next, multivariate Cox regression analysis was conducted to further identify independent prognostic RNAs. *p* value < 0.05 tested by log-rank testing was chosen as the statistical threshold. Then, the Cox proportional hazards model (least absolute shrinkage and selection operator (LASSO)) based on the L1-penalized regularization regression algorithm in the penalized package (version, 0.9-5; http://bioconductor.org/packages/penalized/) [21, 22] was used to obtain the optimal RNA combination. Finally, the prognostic risk score was built according to the expression levels of prognostic RNAs (Exp_DERs_) and their prognostic coefficients (∑*β*_DERs_), with the calculation formula as follows:

Prognostic risk score = ∑*β*_DERs_ × Exp_DERs_.

### 2.5. Performance Assessment of the Prognostic Classifier

ALL patients were classified into the high-risk and low-risk subgroups according to the median cut-off of the prognostic risk score. The prognostic performance was evaluated by plotting the Kaplan-Meier survival curve and receiver operating characteristic (ROC) curve using the corresponding function in R statistical software (version 3.4.1; https://cran.r-project.org/). These analyses were first performed for the training dataset (TARGET) and then for validation datasets (E-MTAB-1216, E-MTAB-1205). To validate if the risk score was an independent prognostic factor, univariate and multivariate Cox regression analyses were performed based on the risk score and various clinical features in the training dataset, with *p* value < 0.05 considered statistically significant. Furthermore, the predictive power of this prognostic classifier for the probability of the relapse was also explored in all datasets by calculating accuracy, sensitivity, specificity, positive predictive value (PPV), and negative predictive value (NPV) from the 2 × 2 confusion matrix [23] and ROC curve analysis using the pROC package in R (v1.14.0; https://cran.r-project.org/web/packages/pROC/index.html).

### 2.6. Function Enrichment Analyses of the Prognostic RNAs

To detailedly clarify how these prognostic RNAs influence the related clinical outcomes, function enrichment analyses were performed. The functions of lncRNAs were estimated according to their interactions with protein-encoding mRNAs in crucial modules. Thus, a coexpression network was constructed based on the Pearson correlation coefficients (PCC) between lncRNAs and mRNAs which were calculated using the cor.test function (https://stat.ethz.ch/R-manual/R-devel/library/stats/html/cor.test.html) in R. The network was visualized in the Cytoscape software (version 3.6.1; http://www.cytoscape.org/). Then, the genes in the coexpression network were uploaded to the online tool Database for Annotation, Visualization, and Integrated Discovery (DAVID) (version 6.8; http://david.abcc.ncifcrf.gov) [24] to accomplish function enrichment analysis. Gene Ontology (GO) biological process terms and Kyoto Encyclopedia of Genes and Genomes (KEGG) and REACTOME pathways were collected to indicate the functions of genes. A *p* value < 0.05 was considered statistically significant.

## 3. Results

### 3.1. Identification of Important Coexpression Modules in Relapsed ALL

**Figure 1:**
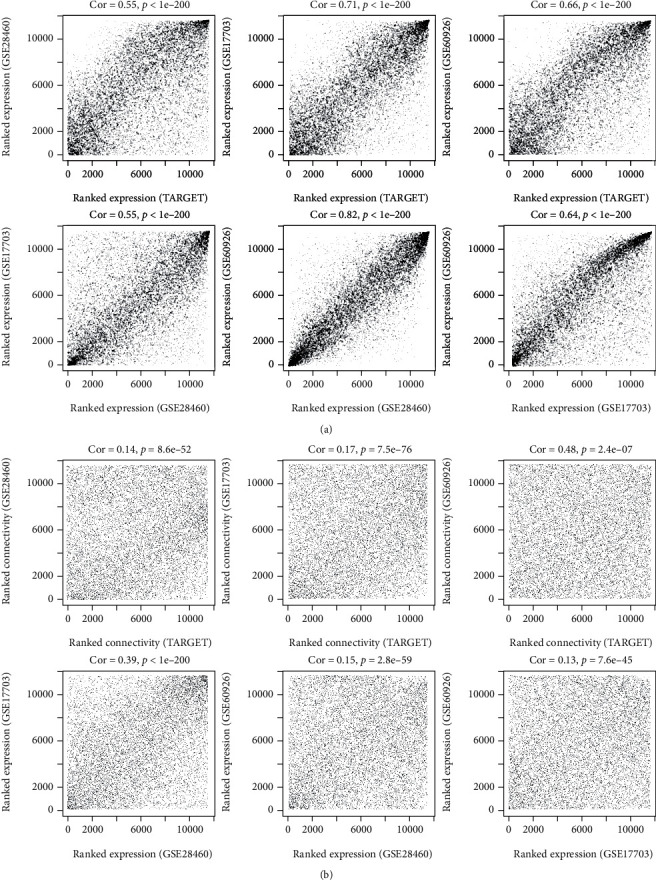
The correlation between any two datasets (TARGET, GSE60926, GSE28460, and GSE17703) in the RNA expression levels (a) and connectivity (b).

**Figure 2:**
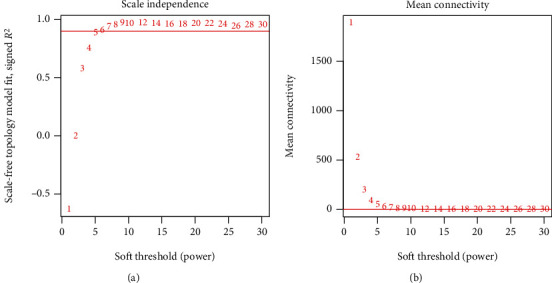
Selection graphs of the soft-thresholding power *β* in the adjacency matrix (a) and schematic diagram of the mean connectivity of RNA under various power values (b).

**Figure 3:**
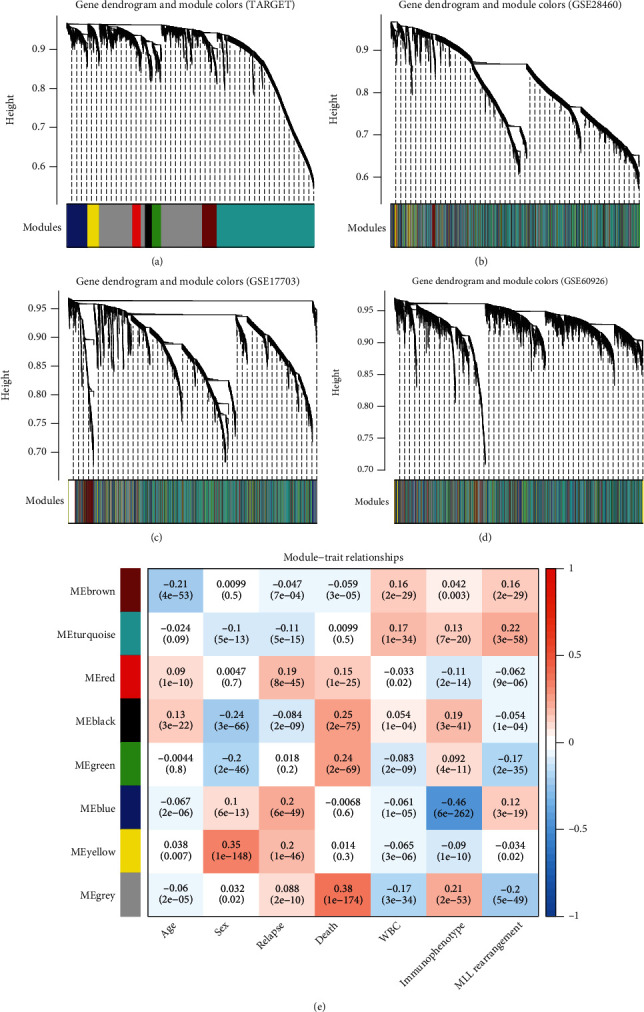
Clustering dendrograms of gene modules screened from the datasets TARGET (a), GSE28460 (b), GSE17703 (c), and GSE60926 (d) and the association between functional modules of RNAs in the TARGET dataset and the clinical characteristics of acute lymphoblastic leukemia patients (e). In the module-trait heat map, each column corresponds to clinical parameters and each row corresponds to a module eigengene. The correlation coefficients are shown at the top of each row. The corresponding *p* values for each module are displayed at the bottom of each row within parentheses. WBC: white blood cell; MLL: mixed lineage leukemia.

After HGNC annotation and comparison, 97 lncRNAs and 11,488 protein-encoding mRNAs were found to be shared in all included datasets which were used for the WGCNA analysis. As shown in Figure 1, the correlation coefficient of any two datasets in the RNA expression levels was larger than 0.5 and the statistical *p* value was less than 1*e* − 200 (Figure 1(a)). Additionally, the correlations for connectivity were also positive, and the *p* values were significant between any two datasets (Figure 1(b)). These findings suggested that our included four datasets for WGCNA analysis were comparable. When the soft-thresholding power *β* was set to 6, *R*^2^ reached 0.9 for the first time (Figure 2(a)) and the average connection degree of RNAs was equal to 1 (Figure 2(b)), indicating at this time that the network met a scale-free distribution and had a small-world characteristic. Using the DynamicTreeCut method, eight coexpression gene modules (Table 1) were extracted using the training TARGET dataset (Figure 3(a)), among which the turquoise module contained the most genes (1,980 mRNA and 28 lncRNAs). These modules seemed to still group together in other three validation datasets (Figures 3(b)–3(d)), suggesting that these modules may possess good preservation. In order to further confirm the stability of these modules, modulePreservation statistical analysis was performed. As a result, three modules showed a *Z*-score between 5 and 10 (green, turquoise, and yellow), while the *Z*-score was larger than 10 in one module (brown) (Table 1), implying that the genes in these four modules exhibiting moderate-high preservation may be especially crucial for the development of ALL. This conclusion can also be seen from the association analysis results between the module genes and clinical features (Figure 3(e)), in which the brown and green modules were significantly associated with patients' death, while brown, turquoise, and yellow were significantly related to patients' relapse status.

### 3.2. Identification of Differentially Expressed Module RNAs in Relapsed ALL

**Figure 4:**
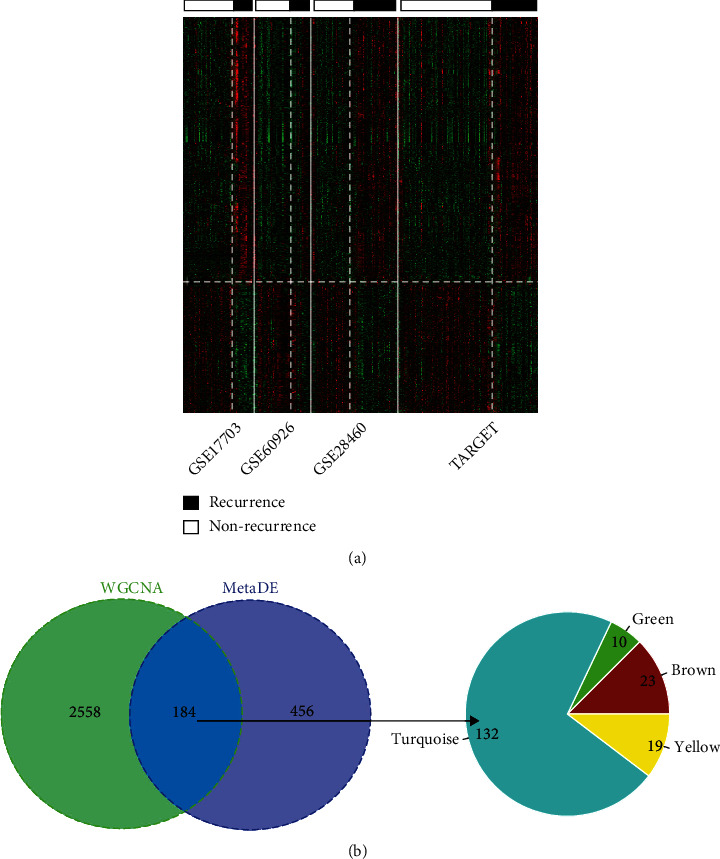
Identification of differentially expressed module genes. (a) Heat map of differentially expressed RNAs; (b) Venn diagram to show the overlap between differentially expressed RNAs and module genes.

According to three inclusion criteria (tau^2^ and *Qp* value > 0.05, FDR < 0.05, and similar pattern of FC), a total of 640 RNAs were found to be differentially expressed in four datasets (TARGET, GSE60926, GSE28460, and GSE17703), including 321 downregulated and 319 upregulated. The heat map showed that these 640 DERs can obviously distinguish the recurrence from the nonrecurrence samples in each dataset (Figure 4(a)). Subsequently, these 640 genes were overlapped with 2,742 genes of the above-identified four crucial modules. As a result, only 184 (including 8 lncRNAs and 176 mRNAs) were found to be common, 23 of which belonged to the brown module, 132 of the turquoise module, 10 of the green module, and 19 of the yellow module (Figure 4(b)), suggesting that they represent potential biomarkers for ALL.

### 3.3. Identification of a Prognostic Signature from Module RNAs

Univariate Cox regression analysis was performed for these 184 module RNAs to preliminarily identify prognostic biomarkers. The results showed that 23 of them (19 mRNAs and 4 lncRNAs: ADD3-AS1, IGF2-AS, LINC00652, and LINC00588) were significantly associated with OS, with a log-rank *p*-value less than 0.05. Then, these 23 RNAs underwent additional multivariate Cox regression analysis to validate their prognostic independence, which resulted in 9 obtained (6 mRNAs and 3 lncRNAs: ADD3-AS1, IGF2-AS, and LINC00652). LASSO was next applied to screen the optimal RNA combination, ultimately leading to a prognostic signature constructed by 7-RNA to be selected (Table 2). These 7 RNAs were derived from turquoise, green, and yellow modules (Table 2), which were in line with the module-clinical phenotype relationship analysis (Figure 3(e)).

**Figure 5:**
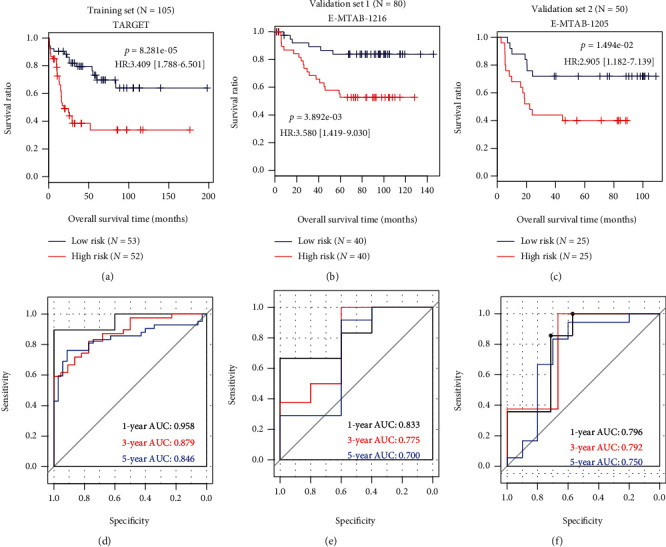
The prognostic performance of the risk score model established by the seven-lncRNA-mRNA signature genes. (a) Kaplan-Meier survival curve of the training dataset, TARGET; (b) Kaplan-Meier survival curve of validation dataset 1, E-MTAB-1216; (c) Kaplan-Meier survival curve of validation dataset 2, E-MTAB-1205; (d) ROC of the training dataset, TARGET; (e) ROC curve of validation dataset 1, E-MTAB-1216; (f) ROC curve of validation dataset 2, E-MTAB-1205. HR: hazard ratio; ROC: receiver operator characteristic curve; AUC: area under the ROC curve.

**Figure 6:**
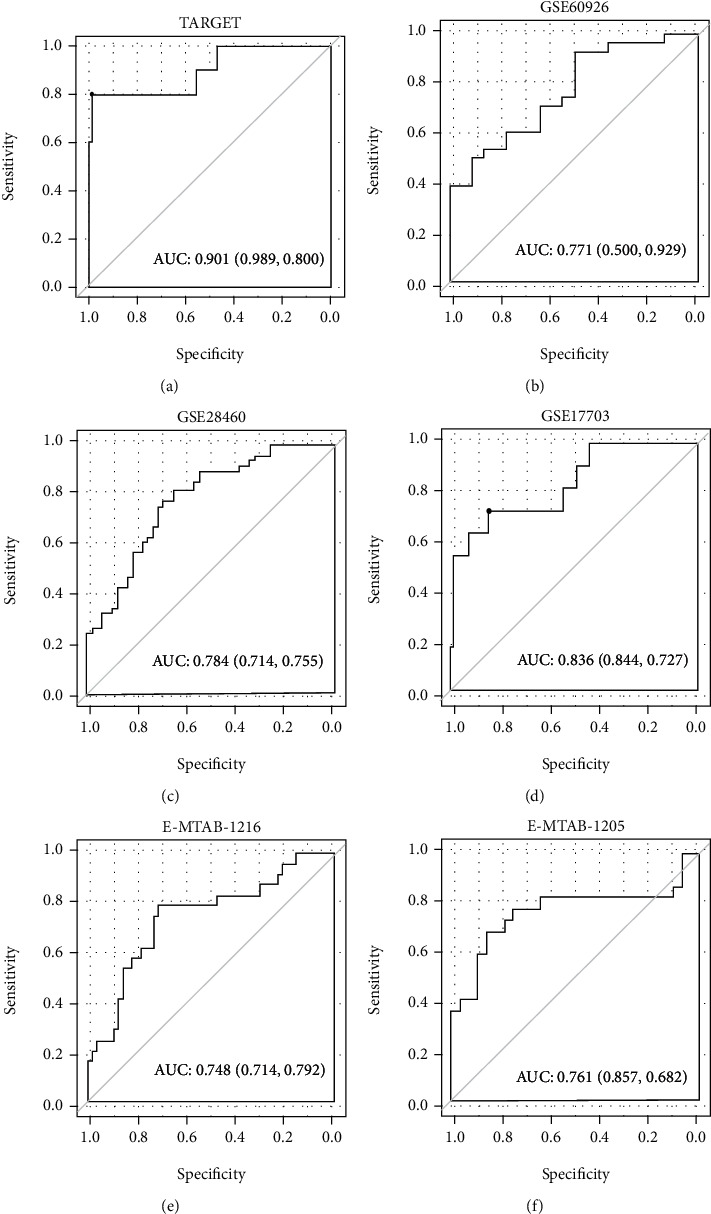
The predictive performance of the seven-lncRNA-mRNA signature for recurrence outcomes in different datasets: (a) ROC curve of TARGET; (b) receiver operator characteristic curve of GSE60926; (c) ROC of GSE28460; (d) ROC curve of GSE17703; (e) ROC curve of E-MTAB-1216; (f) ROC curve of E-MTAB-1205. ROC: receiver operating characteristic curve; AUC: area under the ROC curve.

The prognostic score was calculated by these 7 RNAs according to the following formula: (−0.00432 × expression of LINC00652) + (0.00739 × expression of INSL3 [insulin − like 3]) + (0.00362 × expression of NIPAL2 [NIPA − like domain containing 2]) + (0.02222 × expression of REN [renin]) + (0.00769 × expression of RIMS2 [regulating synaptic membrane exocytosis 2]) + (0.01680 × expression of RPRM [reprimo, TP53 dependent G2 arrest mediator homolog]) + (0.02990 × expression of SNAP91 [synaptosome associated protein 91]). According to the corresponding median cut-off, the patients in the training and validation datasets were separated into the low-risk and high-risk subgroups. Kaplan-Meier curve analysis in all datasets showed that patients in the high-risk group had a significantly shorter OS than those of the low-risk group (training TARGET: hazard ratios (HR) = 3.409, 95%confidence intervals (95%CI) = 1.788‐6.501, *p* = 8.281*e* − 05, Figure 5(a); validation E-MTAB-1216: HR = 3.580, 95%CI = 1.419‐9.030, *p* = 3.892*e* − 03, Figure 5(b); and validation E-MTAB-1205: HR = 2.905, 95%CI = 1.1829‐7.139, *p* = 1.494 − 02, Figure 5(c)). ROC curve analysis also demonstrated that this signature had high prediction accuracy for OS, with the 1-, 3-, and 5-year areas under the curve (AUC) of 0.958, 0.879, and 0.846 in TARGET (Figure 5(d)); 0.833, 0.775, and 0.700 in E-MTAB-1216 (Figure 5(e)); and 0.796, 0.792, and 0.750 in E-MTAB-1205 (Figure 5(f)), respectively. Furthermore, univariate and multivariate Cox regression analyses were also performed to investigate whether our model was a clinically independent prognostic factor. The results showed that age, WBC at diagnosis, immunophenotype, MLL rearrangement, and the risk score were significant factors to be associated with OS in univariate analysis, while only age and the risk score were identified to be independent prognostic factors after multivariate analysis (Table 3). More importantly, the prognostic classifier was observed to provide high predictive power for the recurrence status in all datasets (TARGET: AUC = 0.901, Figure 6(a); GSE60926: AUC = 0.771, Figure 6(b); GSE28460: AUC = 0.784, Figure 6(c); GSE17703: AUC = 0.836, Figure 6(d); E-MTAB-1216: AUC = 0.748, Figure 6(e); and E-MTAB-1205: AUC = 0.761, Figure 6(f)). The accuracy, sensitivity, specificity, PPV, and NPV are shown in Table 4.

### 3.4. Functional Annotation of Prognostic Genes

**Figure 7:**
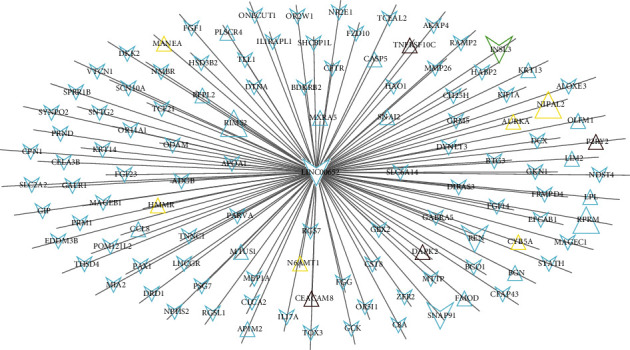
A coexpression network between prognostic LINC00652 and its differentially expressed mRNAs. Triangle indicates the upregulated RNAs; inverted triangle indicates the downregulated RNAs; the color of nodes is corresponding to the module color (turquoise, green, yellow, and brown); the nodes with larger font are prognostic signature RNAs.

To explore the functional involvement of the prognostic genes, a coexpression network was constructed and DAVID enrichment analysis was then performed. Based on the cut-off point of PCC > 0.4, 113 coexpression pairs (Figure 7) were collected between prognostic LINC00652 and its target genes (including all the prognostic protein-encoding mRNAs (INSL3, PCC = 0.51; NIPAL2, PCC = 0.43; REN, PCC = 0.471; RIMS2, PCC = 0.57; RPRM, PCC = 0.62; and SNAP91, PCC = 0.86)). All these 113 target genes of LINC00652 in the coexpression network were uploaded into DAVID, and 41 genes of them (including three prognostic genes: INSL3, REN, and RIMS2) were enriched into function results, such as GO:0006508~proteolysis (REN), hsa04911: insulin secretion (RIMS2), and R-HSA-418555: G alpha (s) signaling events (INSL3) (Table 5). Although the other prognostic genes were not enriched by DAVID, we speculated that they (except NIPAL2) may be involved in ALL similar to the enriched genes because all these genes belonged to the same (turquoise) module as REN and RIMS2 and the functions of green and turquoise modules were shown to be similar in userListEnchment analysis (Table 1). NIPAL2 may be involved in mitosis according to the results of userListEnchment analysis (Table 1). Interestingly, melanoma inhibitory activity 2 (MIA2) and apolipoprotein A1 (APOA1) were enriched into the top function result according to *p* value ranking, such as GO:0070328~triglyceride homeostasis (MIA2, APOA1), GO:0042632~cholesterol homeostasis (MIA2, APOA1), and R-HSA-174800: chylomicron-mediated lipid transport (APOA1), suggesting that they may also be crucial downstream targets for LINC00652 to participate in the development of ALL.

## 4. Discussion

Although there are studies that attempt to develop a prognostic risk scoring system of lncRNAs for leukemia patients [25–27], all of them focused on the type of acute myeloid leukemia (AML) and did not specifically investigate the childhood population. Also, a previous study indicated that the predictive accuracy seemed to be better using the integrated mRNA-lncRNA signature (AUC = 0.791) than the mRNA (AUC: 0.584) or lncRNA alone (AUC: 0.527) [28]. Therefore, in this study, we aimed to, for the first time, identify an lncRNA-mRNA prognostic signature for relapsed childhood ALL patients. As a result, a seven-gene-based risk score (including 1 lncRNA: LINC00652 and 6 mRNAs: INSL3, NIPAL2, REN, RIMS2, RPRM, and SNAP91) was constructed. ROC curve analysis demonstrated that this prognostic signature exhibited good performance for predicting 1-, 3-, and 5-year OS of childhood ALL patients of both the training and two validation datasets, with the AUC ranging from 0.796 to 0.958 for 1-year OS, 0.775 to 0.879 for 3-year OS, and 0.700 to 0.846 for 5-year OS, respectively. In line with the study of Xiang et al. [28], the prognostic accuracy of our integrated lncRNA-mRNA signature screened from the training dataset also seemed to be higher than that of the study performed by Chang et al. (AUC: 0.846 vs. 0.7984, only including 15 apoptosis pathway genes) [29]. Likewise, the predictive power of this lncRNA-mRNA signature for recurrence outcomes was also higher than that of the mRNA signature identified by Cleaver et al. [13] (five-mRNA classifier: accuracy: 93.3% vs. 82%, PPV: 94.7% vs. 81%, and sensitivity: 97.8% vs. 77%; 7-NF-*κ*B pathway genes: accuracy: 93.3% vs. 76%, PPV: 94.7% vs. 71%, and sensitivity: 97.8% vs. 77%; 12-Wnt pathway genes: accuracy: 93.3% vs. 76%, PPV: 94.7% vs. 75%, NPV: 80.0% vs. 77%, and sensitivity: 97.8% vs. 68%; and 14-cell adhesion pathway genes: accuracy: 93.3% vs. 82%, PPV: 94.7% vs. 84%, and sensitivity: 97.8% vs. 73%).

There were rare studies that investigate the roles of LINC00652, except one that analyzed its functions in myocardial ischemia-reperfusion injury: LINC00652 was found to be overexpressed in the myocardial cells of mice with myocardial ischemia-reperfusion injury. Knockdown of LINC00652 suppressed cardiac pathology, infarct size, and apoptosis rates of myocardial cells [30]. These findings indirectly reveal the possible proapoptotic effects of LINC00652. Thus, theoretically, LINC00652 may be downregulated in cancer. This hypothesis was confirmed in our differential analysis between recurrent and nonrecurrent samples of four datasets (TARGET, GSE17703, GSE28460, and GSE60926). Also, multivariate Cox regression analysis showed that a high LINC00652 level was a protective factor for OS (HR, 0.980; 95% CI, 0.949-0.993, *p* = 2.30*e* − 02). However, its mechanism in cancer remains unclear. In this study, we predicted that downregulated LINC00652 may positively correlate with several downregulated prognostic genes (INSL3, SNAP91, and REN) to influence the development of childhood ALL. These genes had been demonstrated to be related to cancers, which may indirectly verify our prediction for the roles of LINC00652. For example, Rossato et al. used the immunohistochemistry analysis to demonstrate that INSL3 expression was negative or decreased in Leydig cell tumor samples, but strongly and diffusely positive in normal Leydig cells and Leydig cell hyperplasia [31]. Lottrup et al. also confirmed that INSL3 could not be detectable in testicular adrenal rest tumors [32]. Kaplan-Meier analysis of SNAP91 in the GSE7696 dataset showed that glioblastoma patients with high expression of SNAP91 exhibited a higher survival ratio compared with those having low levels of SNAP91 [33]. Quantitative real-time PCR revealed that SNAP91 was lowly expressed in human esophageal cancer tissues and cell lines. Low expression of SNAP91 was associated with poor prognosis in patients with esophageal cancer [34]. In accordance with these studies, we also validated that both INSL3 and SNAP91 were downregulated in recurrent ALL samples. However, the survival analysis of INSL3 and SNAP91 indicated that they may be oncogenes and were risk factors for a poor prognosis. This result seemed to be believable because *in vitro* and *in vivo* studies on thyroid cancer demonstrated that recombinant and secreted INSL3 increased the motility and growth of thyroid carcinoma cells and enhanced the formation of fast-growing and highly vascularized xenografts in nude mice [35]. INSL3 was also demonstrated to promote early tumor cell invasiveness in human thyroid carcinoma cells by enhancing their metabolic activity and elastin-degrading potential via increasing the production of the lysosomal enzymes cathepsin-L and cathepsin-D [36]. Nevertheless, the roles of INSL3 and SNAP91 in ALL cells still need further experimental investigation in the future. There was evidence to demonstrate that the disappearance of REN expression was associated with the status of CR in ALL patients [37] and improved survival [38]. The use of renin-angiotensin system inhibitor losartan induced the apoptosis of leukemic cells [39]. These results reflected the possible positive association between a high expression level of REN and poor OS in ALL patients. This theory was validated in our study (HR > 1).

In addition to prognostic genes, we also found a positive coexpression relationship between LINC00652 and MIA2/APOA1. These two genes were enriched into the top function result (lipid homeostasis triglyceride cholesterol), and thus, they may also be underlying downstream targets for LINC00652 to influence the development of ALL. MIA2 encodes a receptor in the endoplasmic reticulum, which plays a role in the export of large prechylomicrons and pre-very-low-density lipoproteins (pre-VLDLs). The low expression of MIA2 may result in the accumulation of VLDLs which was reported to be a risk factor for the development of cancer [40, 41]. Therefore, MIA2 may be downregulated in cancer. This assumption had been demonstrated in hepatocellular carcinoma [42, 43] and gastric cancer [44]. Treatment with recombinant MIA2 inhibited proliferation and invasion of hepatocellular carcinoma cells *in vitro* and *in vivo*. Loss of MIA2 expression significantly correlated with advanced tumor stages [42]. APOA1 is the major protein in high-density lipoprotein (HDL). HDL is a beneficial protein in humans, and its low level was shown to be associated with an increased risk of cancer [40]. Hereby, APOA1 expression was also inversely associated with cancer risk, which was proved in lung cancer [45]. Treatment with APOA1 and the mimetic peptide was reported to decrease the viability and prevented cell invasion of ovarian cancer [46]. Meta-analysis showed that lower APOA1 was associated with unfavorable cancer-specific survival and shorter DFS in overall cancer, inferior total time to recurrence in hepatocellular carcinoma, poorer locoregional RFS, and distant metastasis-free survival in nasopharyngeal carcinoma [47]. In line with these findings, we also identified that MIA2 and APOA1 were downregulated in recurrent ALL samples. These findings indicated that LINC00652 may also be involved in the progression of ALL by regulating the balance between LDL and HDL via influencing the expressions of MIA2 and APOA1.

The function of RIMS2 in ALL was also not explored previously and could only be indirectly reflected by the studies on other cancers. After analysis of The Cancer Genome Atlas cases, Ke et al. [48] and Wu et al. [49] identified RIMS2 as a prognostic gene for papillary thyroid carcinoma and lung adenocarcinoma patients, respectively. Patients with a high expression level of RIMS2 were reported to have a higher probability of survival compared with cases with a low expression level [48, 49]. Immunoprecipitation experiments revealed that RIMS2 may promote anchorage-independent growth and colony formation of liver metastatic breast cancer cells by binding with claudin-2 gene via a PDZ-binding motif [50]. Consistent with these findings, we also identified that RIMS2 was upregulated in recurrent ALL samples and high expression of RIMS2 was associated with poor OS.

Until now, no attempt had been made to study NIPAL2. Immunostaining observed that its family member NIPAL1 was expressed in 20.3% of oral squamous cell carcinoma patients. High NIPAL1 expression significantly correlated with cancer cell intravasation and poorer disease-free survival. siRNA-mediated knockdown of NIPAL1 significantly inhibited the growth and adhesion of oral squamous cell carcinoma cells compared with negative siRNA [51]. These findings indicated that NIPAL2 may also be a tumor-promoting factor in ALL as NIPAL1. This hypothesis was confirmed in our study, with expression of upregulation in recurrent ALL samples and HR > 1 in prognostic analysis.

There were some limitations in this study. First, relative to AML, the studies on ALL were rare, and thus, the sample size was small in most of the datasets (<100). This may be a potential reason to lead to the expression difference in partial genes compared with the published literatures (RPRM was found to be downregulated in cancers due to promoter methylation [52–54], but upregulated in our ALL). Also, our used data were retrospectively collected from TARGET and EMBL-EBI public databases, and some clinical information was not described. Thus, larger clinical trials should be prospectively designed in our hospital to validate the expression of all our prognostic genes, their prognostic value (alone or combined form), and association with genetic subtypes in ALL cohorts via quantitative PCR. Second, the coexpression relationship between LINC00652 and INSL3/SNAP91/MIA2/APOA1 should be verified by coimmunoprecipitation experiments. Third, the effects of our identified lncRNAs and mRNAs on the proliferation, apoptosis, metastasis, and invasion of childhood ALL cell lines need to be explored by overexpression and knockout experiments *in vitro* and *in vivo*.

## 5. Conclusion

In the present study, we developed a new lncRNA-mRNA signature (including lncRNA LINC00652 and its six coexpression genes, INSL3, NIPAL2, REN, RIMS2, RPRM, and SNAP91) in relapsed ALL patients based on WGCNA and LASSO analyses. This classifier had high accuracy in predicting 5-year OS (AUC = 0.846) and recurrence outcomes (AUC = 0.901). Therefore, it may be a potential predictive and prognostic biomarker applied in the clinic for childhood ALL.

## Figures and Tables

**Table 1:** Stable modules screened using weighted gene coexpression network analysis.

ID	Color	Module size	Number of mRNAs	Number of lncRNAs	Preservation *Z*-score	Module annotation
Module 1	Black	138	136	2	4.6749	Neurological system process
Module 2	Blue	424	418	6	1.0193	Cell motion
Module 3	Brown	302	302	0	14.1644	Defense response
Module 4	Green	193	191	2	5.3994	Cell-cell signaling
Module 5	Grey	1658	1640	18	2.1860	Cell adhesion
Module 6	Red	174	172	2	2.7514	Ion transport
Module 7	Turquoise	2008	1980	28	8.1713	Cell-cell signaling
Module 8	Yellow	239	236	3	5.1719	Mitosis

**Table 2:** The optimal prognostic signature.

Symbol	Module	Expression	Type	Multivariate Cox regression analysis			LASSO coefficient
				HR	95% CI	*p* value	
LINC00652	Turquoise	Downregulated	lncRNA	0.980	0.949-0.993	2.30*e* − 02	-0.00432
INSL3	Green	Downregulated	mRNA	1.008	1.004-1.012	2.41*e* − 05	0.00739
NIPAL2	Yellow	Upregulated	mRNA	1.004	1.001-1.007	1.20*e* − 02	0.00362
REN	Turquoise	Downregulated	mRNA	1.024	1.004-1.045	1.75*e* − 02	0.02222
RIMS2	Turquoise	Upregulated	mRNA	1.011	1.005-1.033	3.49*e* − 02	0.00769
RPRM	Turquoise	Upregulated	mRNA	1.020	1.006-1.035	6.60*e* − 03	0.01680
SNAP91	Turquoise	Downregulated	mRNA	1.050	1.001-1.101	4.80*e* − 02	0.02990

HR: hazard ratio; CI: confidence interval; LASSO: least absolute shrinkage and selection operator.

**Table 3:** Univariate and multivariate Cox regression analyses of overall survival.

Clinical characteristics	TARGET (*N* = 105)	Univariate Cox		Multivariate Cox	
		HR (95% CI)	*p* value	HR (95% CI)	*p* value
Age (years, mean ± SD)	8.53 ± 5.48	0.967 (0.639-0.993)	2.36*e* − 02	0.965 (0.903-0.989)	3.05*e* − 02
Sex (male/female)	61/44	0.779 (0.429-1.413)	4.10*e* − 01	—	—
WBC at diagnosis (IU, mean ± SD)	96.28 ± 158.33	1.001 (0.999-1.003)	1.44*e* − 02	0.999 (0.996-1.002)	5.75*e* − 01
Relapse (yes/no)	10/95	1.271 (0.534-3.021)	5.87*e* − 01	—	—
Immunophenotype (T/B/mixture)	45/37/23	1.903 (1.260-2.875)	1.78*e* − 03	1.779 (0.941-2.851)	1.68*e* − 01
MLL rearrangement (yes/no)	8/97	3.160 (1.231-8.113)	1.16*e* − 02	1.664 (0.350-7.913)	5.22*e* − 01
Prognostic score model status (high/low)	53/52	3.409 (1.788-6.501)	8.28*e* − 05	3.787 (1.837-7.808)	3.10*e* − 04
Death (dead/alive)	44/61	—	—	—	—
Overall survival (months, mean ± SD)	41.86 ± 39.65	—	—	—	—

SD: standard deviation; WBC: white blood cell; TARGET: Therapeutically Applicable Research to Generate Effective Treatments; HR: hazard ratio; CI: confidence interval; MLL: mixed lineage leukemia.

**Table 4:** Validation of the prognostic classifier models for recurrence prediction.

	Accuracy (%)	Positive predicted value (%)	Negative predicted value (%)	Sensitivity (%)	Specificity (%)
TARGET	93.3	94.7	80.0	97.8	61.5
GSE60926	84	72.7	92.9	88.9	81.3
GSE28460	71.4	67.3	75.5	73.3	69.8
GSE17703	90.1	93.3	63.6	95.5	53.8
E-MTAB-1216	78.8	85.7	62.5	84.2	65.2
E-MTAB-1205	78	82.1	72.7	79.3	76.2

**Table 5:** Function enrichment results.

Category	Term	*p* value	Genes
GO BP	GO:0070328~triglyceride homeostasis	5.11*e* − 04	MIA2, LPL, GIP, APOA1
GO BP	GO:0006508~proteolysis	3.38*e* − 03	ADGB, CASP5, CELA3B, CLCA2, REN, THSD4, MEP1A, MMP26, TLL1, HABP2
GO BP	GO:0042632~cholesterol homeostasis	6.93*e* − 03	MIA2, LPL, APOA1, MTTP
GO BP	GO:0007200~phospholipase C-activating G-protein coupled receptor signaling pathway	7.55*e* − 03	GALR1, P2RY2, LHCGR, NMBR
GO BP	GO:0001662~behavioral fear response	1.33*e* − 02	DRD1, GABRA5, NR2E1
GO BP	GO:0060291~long-term synaptic potentiation	2.23*e* − 02	DRD1, GIP, NR2E1
GO BP	GO:0007189~adenylate cyclase-activating G-protein coupled receptor signaling pathway	3.71*e* − 02	DRD1, GALR1, LHCGR
GO BP	GO:0019233~sensory perception of pain	3.98*e* − 02	GIP, ALOXE3, SCN10A
GO BP	GO:0007186~G-protein coupled receptor signaling pathway	4.64*e* − 02	OR5I1, RAMP2, FZD10, APOA1, LHCGR, CCL8, RGS7, OR11A1, BDKRB2, NMBR, OR2W1
KEGG	hsa04080: neuroactive ligand-receptor interaction	4.50*e* − 03	GRM5, DRD1, GALR1, P2RY2, GABRA5, LHCGR, BDKRB2, NMBR
KEGG	hsa04950: maturity onset diabetes of the young	1.62*e* − 02	ONECUT1, GCK, SLC2A2
KEGG	hsa04911: insulin secretion	2.61*e* − 02	GIP, GCK, SLC2A2, RIMS2
KEGG	hsa04020: calcium signaling pathway	4.56*e* − 02	GRM5, DRD1, TNNC1, LHCGR, BDKRB2
REACTOME	R-HSA-174800: chylomicron-mediated lipid transport	1.12*e* − 02	LPL, APOA1, MTTP
REACTOME	R-HSA-418555: G alpha (s) signaling events	1.54*e* − 02	RAMP2, INSL3, DRD1, GIP, LHCGR
REACTOME	R-HSA-416476: G alpha (q) signaling events	2.473*e* − 02	GRM5, P2RY2, RGSL1, BDKRB2, NMBR
REACTOME	R-HSA-975634: retinoid metabolism and transport	3.36*e* − 02	LPL, APOA1, BCO1

KEGG: Kyoto Encyclopedia of Genes and Genomes; GO BP: Gene Ontology biological process.

## Data Availability

The normalized RNA-seq or array data were downloaded from the TARGET (https://ocg.cancer.gov/programs/target) and EMBL-EBI (https://www.ebi.ac.uk/arrayexpress/) databases.
